# Methadone in pain management: About a case and a review of the literature

**DOI:** 10.1192/j.eurpsy.2023.2048

**Published:** 2023-07-19

**Authors:** F. Azraf, H. Berrada, M. Sabir, F. El Omari

**Affiliations:** 1 Arrazi hospital; 2 Faculty of Medicine and Pharmacy of Rabat, sale, Morocco

## Abstract

**Introduction:**

Methadone has been available for about half a century. It is traditionally known for its role in the withdrawal and maintenance of patients addicted to heroin or other opioids.

A large body of evidence has identified a number of advantages of methadone over other opioids in the treatment of pain, including its agonist action at μ- and δ-opioid receptors, its N-methyl-D-aspartate (NMDA) antagonist activity, and the ability to inhibit monoamine reuptake, hence its interest in the treatment of cancer pain and, more recently, neuropathic pain and non-cancer pain.

Methadone proves to be a treatment adapted to the management of complex painful situations, resistant to other opioids. It sometimes makes it possible to postpone an invasive act, sedation for refractory symptoms while maintaining the patient’s autonomy. Its use in many countries and feedback from our colleagues agree with our observations. It is an effective molecule as an analgesic on different types of pain, including post-surgical pain, cancer-related pain or nociceptive pain

**Objectives:**

explain the role of methadone in the treatment of chronic and acute pain.

**Methods:**

We will explain through a clinical case the role of methadone in the treatment of chronic and acute pain. The patient was a 35-year-old nurse, married with 3 children, with problematic use of codeine and morphine for chronic pain due to endometriosis. She was put on 20mg/d of methadone with good clinical improvement.

**Results:**

We reported the clinical case of a patient followed for endometriosis and that she presents with acute and chronic pain during and outside of menstruation. The patient was treated with (Danazol) and analgesics to manage her pain. She was initially put on level 1 analgesics: paracetamol and NSAIDs, then on level 2 analgesics, in particular codeine at a rate of 200 mg/day, without any improvement. Faced with this state, the patient was put on morphine with a gradual increase in doses on her own initiative up to 30mg/d. The patient tried to stop her morphine consumption on several occasions without succeeding. The patient was put on methadone to treat both her pain and her addiction to morphine, methadone significantly reduced her pain within a few days.

**Conclusions:**

Methadone proves to be a treatment adapted to the management of complex painful situations, resistant to other opioids. It sometimes makes it possible to postpone an invasive act, sedation for refractory symptoms while maintaining the patient’s autonomy. Its use in many countries and feedback from our colleagues agree with our observations. It is an effective molecule as an analgesic on different types of pain, including post-surgical pain, cancer-related pain or nociceptive pain.

**Disclosure of Interest:**

None Declared

